# Vascular Endothelial Growth Factor-A Exerts Diverse Cellular Effects via Small G Proteins, Rho and Rap

**DOI:** 10.3390/ijms19041203

**Published:** 2018-04-16

**Authors:** Akio Shimizu, Dimitar P. Zankov, Misuzu Kurokawa-Seo, Hisakazu Ogita

**Affiliations:** 1 Division of Molecular Medical Biochemistry, Department of Biochemistry and Molecular Biology, Shiga University of Medical Science, Otsu, Shiga 520-2192, Japan; shimizua@belle.shiga-med.ac.jp (A.S.); dzankoff@belle.shiga-med.ac.jp (D.P.Z.); 2 Department of Molecular Biosciences, Faculty of Life Sciences, Kyoto Sangyo University, Kyoto 603-8555, Japan; mseo@cc.kyoto-su.ac.jp

**Keywords:** angiogenesis, cell adhesion, cell migration, cell proliferation, growth factor, small G protein, tumor progression

## Abstract

Vascular endothelial growth factors (VEGFs) include five molecules (VEGF-A, -B, -C, -D, and placental growth factor), and have various roles that crucially regulate cellular functions in many kinds of cells and tissues. Intracellular signal transduction induced by VEGFs has been extensively studied and is usually initiated by their binding to two classes of transmembrane receptors: receptor tyrosine kinase VEGF receptors (VEGF receptor-1, -2 and -3) and neuropilins (NRP1 and NRP2). In addition to many established results reported by other research groups, we have previously identified small G proteins, especially Ras homologue gene (Rho) and Ras-related protein (Rap), as important mediators of VEGF-A-stimulated signaling in cancer cells as well as endothelial cells. This review article describes the VEGF-A-induced signaling pathways underlying diverse cellular functions, including cell proliferation, migration, and angiogenesis, and the involvement of Rho, Rap, and their related molecules in these pathways.

## 1. Introduction

Vascular endothelial growth factors (VEGFs) are secreted glycoproteins. There are five VEGF family members in mammals: VEGF-A through D, and placental growth factor (PlGF) [1]. The three distinct VEGF receptors (1 through 3) harbor tyrosine kinase activity in their cytoplasmic regions [2]. The binding of VEGF to a receptor induces receptor dimerization and kinase activation, resulting in the transduction of signals that direct endothelial cell functions. In particular, VEGF-A plays an important role in embryonic vascular development as a regulator of both vasculogenesis and angiogenesis. *Vegf-a* knockout mice show embryonic lethality between E10 and E11 with immature blood vessel formation [3,4]. Even disruption of a single *Vegf-a* allele in mice results in embryonic lethality between E11 and E12, indicating that the local concentration of VEGF-A in the embryo has to be tightly regulated for proper angiogenesis. In adults, overexpression of VEGF-A enhances tumor progression by supplying oxygen and nutrients to tumor cells via the newly generated blood vessels [5,6].

VEGF receptors are type I transmembrane proteins that have an extracellular region with seven immunoglobulin-like domains, one transmembrane domain, and a cytoplasmic region. One of the receptors, VEGF receptor-2 (also named KDR or Flk-1), is preferentially expressed in vascular endothelial cells and transduces various angiogenic signals in response to VEGF-A [7]. After VEGF-A binding, VEGF receptor-2 kinase activity is induced to autophosphorylate tyrosine residues in the intracellular domain. This reaction triggers angiogenic signals, activating a cascade of signaling molecules, including small G proteins [8]. Small G proteins, with masses of 21 to 30 kDa, are monomeric guanine nucleotide-binding proteins. Generally, they stimulate their downstream targets when bound to GTP, but are inactive when bound to GDP [9]. Various small G proteins participate in the events associated with physiological angiogenesis and vascular diseases [10].

In this review, we introduce the functional relationships among VEGF-A, its receptor, and small G proteins. There are more than 150 small G proteins [11], of which we focus on the Ras homologue gene (Rho) and Ras-related protein (Rap) families. The activation of Rho and Rap is regulated by VEGF-A stimulation to exert diverse cellular functions, including migration and proliferation. Understanding the molecular mechanisms that have been revealed by recent research in the field of VEGF-A and small G proteins could be useful for developing novel therapeutics against diseases, such as cancer and cardiovascular disorders.

## 2. Relationship between VEGF-A–VEGF Receptor-2 Signaling and Rho Family Small G Proteins in Endothelial Cells

Among the VEGF receptors, VEGF receptor-2 is considered to be the major mediator of VEGF-A signaling in endothelial cells, and regulates cell proliferation, survival, migration, and permeability [12]. The intracellular signaling pathways downstream of VEGF receptor-2 have been well studied. VEGF receptor-2 impacts on the Raf–extracellular signal-regulated kinase (ERK) pathway to regulate cell proliferation [13]. VEGF receptor-2 also activates phosphoinositide-3 (PI3) kinase, which increases the production of phosphatidylinositol (3,4,5)-trisphosphate (PIP_3_). Increased PIP_3_ contributes to the activation of Akt for cell survival [14], and of endothelial nitric oxide synthase (eNOS) for NO generation and vascular permeability [15]. Phospholipase Cγ binds to phosphorylated Tyr1175 (Tyr1173 in the mouse) of VEGF receptor-2 and mediates the receptor-induced activation of protein kinase C by generation of diacylglycerol and inositol (1,4,5)-trisphosphate, increasing the concentration of intracellular Ca^2+^ [16]. Other signaling molecules, including p38 mitogen-activated protein (MAP) kinase, focal adhesion kinase, and Src, are activated by VEGF-A and contribute to angiogenesis in endothelial cells [17,18,19]. The regulation and function of these signaling pathways are directly or indirectly associated with small G proteins [20,21,22,23].

The VEGF-A–VEGF receptor-2 axis regulates the activation of Rho family members, such as RhoA, Rac1, and Cdc42 [8,24,25]. Rac1 is implicated in regulating VEGF-A-induced vascular permeability and cell migration [26]. Because Rac1 acts together with components of NADPH oxidase, it also controls the generation of reactive oxygen species (ROS) by VEGF-A [27,28]. VEGF-A promotes the phosphorylation of IQGAP1, which forms a complex with Rac1 to stimulate ROS production and enhance endothelial cell migration and proliferation. IQGAP1 bound to activated Rac1 stabilizes it and increases the GTP-bound active form of Rac1 [29]. Conditional knockout of Rac1 under control of the *Tie-2* promoter in mice causes embryonic lethality with severe defects in the development of major blood vessels [30]. These results suggest that Rac1 plays a central role in endothelial cell proliferation, migration, permeability, and angiogenesis in response to VEGF-A.

Phosphorylation of Tyr1214 (Tyr1212 in the mouse) in VEGF receptor-2 has been implicated in VEGF-induced actin remodeling through activation of Cdc42, which is a major regulator of filopodium formation [31]. Human microvascular endothelial cells overexpressing Cdc42GAP, a suppressor of Cdc42, show reduced tubule-forming capacity, whereas knockdown of Cdc42GAP increases tube formation [32]. Mice with *Tie2*-driven endothelium-specific knockout of Cdc42 do not survive to birth because of impaired vessel formation [33,34]. As a molecule downstream of VEGF-A, Cdc42 seems to be important for the development of vascular vessels.

Similar to Rac1 and Cdc42, RhoA plays an essential role in vascular endothelial cell functions downstream of VEGF-A and VEGF receptor-2. RhoA and its effector, Rho kinase (ROCK), attenuate endothelial barrier function by regulating myosin light chain (MLC) phosphorylation via MLC phosphatase [25]. RhoA activation contributes to VEGF-induced hyperpermeability in the endothelium [35]. This is a result of the destabilization of endothelial junctional proteins, such as VE-cadherin. After being activated by VEGF-A through VEGF receptor-2, Src phosphorylates VE-cadherin, weakening its adhesion activity [36]. In parallel, activated Src induces the activation of RhoA to cause actin bundle rearrangement (stress fiber formation), which robustly increases contractile force, resulting in the retraction (shrinkage) of endothelial cells and the disruption of intercellular junctions. Principal junctional apparatuses in endothelial cells are adherens junctions and tight junctions. Adherens junctions are fundamental organizers of cell–cell adhesion complexes, including VE-cadherin. The formation of tight junctions usually occurs after the organization of adherens junctions, although clear separation of adherens junctions from tight junctions is not observed in endothelial cells unlike in epithelial cells [37,38]. This is probably due to the difference of cell height between endothelial cells and epithelial cells, especially columnar epithelial cells in the intestine. Because of the mixed existence of adherens and tight junctional structures at the intercellular interface in endothelial cells, obvious alterations of junctional components or disturbance of topology of the endothelial layer is difficult to detect in the course of VEGF-induced permeability. This may be different from the recent results observed in some distinct types of epithelial barrier dysfunction [39].

The formation of stress fibers to facilitate cellular contraction is also crucial for endothelial cell migration. This phenomenon is tightly regulated by VEGF-A–VEGF receptor-2-induced activation of the RhoA pathway [40]. Furthermore, activation of RhoA by VEGF-A increases the degradation of p27^kip1^, a cyclin-dependent kinase inhibitor, in the G_1_ phase of endothelial cells, resulting in the promotion of cell cycling and proliferation [41]. The assembly of actin fibers for the formation of contractile ring is important for constricting and dividing the cell to produce two daughter cells. Inhibition of RhoA prevents the actin fiber assembly [42]. These results imply a contribution of RhoA to VEGF-A-initiated proliferative signals.

## 3. Pathological Implications of VEGF-A-Induced Rho Activation

As described above, VEGF-A enhances angiogenesis and permeability in the vasculature at least partly through the activation of Rho. Hyperpermeability and angiogenesis are major characteristics of vitreoretinal diseases, including diabetic retinopathy and age-related macular degeneration. VEGF-A and Rho-related signals are considered to play a role in these diseases, because anti-VEGF-A therapy and ROCK inhibitors are effective in preventing their progression [43,44]. Furthermore, several lines of evidence suggest that VEGF-A-initiated signaling to Rho in tumor cells is involved in the promotion of tumor growth and metastasis formation [45,46,47]. Such signaling pathways, in turn, regulate the expression of VEGF-A in tumor cells [48,49]. In addition to VEGF-A, other VEGFs activate Rho-related signals in tumor cells. VEGF-B as well as VEGF-A promote the migration and invasion of colorectal carcinoma cells through VEGF receptor-1 (also named Flt-1) by stimulating ERK as well as c-jun N-terminal kinase to translocate p65, a subunit of nuclear factor-κB, into the nucleus [50]. The signal also sustains the survival of colorectal carcinoma cells and induces epithelial–mesenchymal transition [51]. VEGF-C, another member of the VEGF family, enhances the mobility and invasiveness of several types of cancer cells through VEGF receptor-3 [52]. The VEGF-C–VEGF receptor-3-mediated signal up-regulates Src and p38 MAP kinase activity, which is also elevated by VEGF-A [53,54]. Based on these findings, signaling pathways stimulated by different VEGFs seem to converge inside cancer cells to enhance their migration and invasiveness.

Besides VEGF receptors, neuropilins (NRP1 and NRP2) act as receptors for VEGF-A, but do not have tyrosine kinase activity. The cytoplasmic regions of NRP1 and NRP2 consist of only 44 and 42 amino acids, respectively. NRPs are expressed in cancer cells and contribute to tumor progression [55,56]. NRP1 is strongly expressed in lung, brain, colon, ovarian, and prostate cancers, and this strong expression correlates with poor patient prognosis [57]. VEGF receptor-2 and -3 are hardly expressed in human primary solid cancer cells [58], while the expression level of VEGF receptor-1 varies in cancer cell types [59,60,61]. Thus, in cancer cells, VEGF-A seems to bind mainly to NRP1 for its signal transduction if VEGF receptor-1 expression is low. Inhibition of NRP1 by a blocking antibody prevents tumor growth increased by VEGF-A in a mouse model, suppresses mammosphere formation by breast cancer stem-like cells, and impairs the invasion of glioblastoma [62,63,64]. Rho-related signals are reportedly regulated by NRPs in a VEGF-A-dependent and/or -independent manner [65,66]. VEGF-A activates Akt through NRP1 by an autocrine mechanism to promote breast cancer cell survival [67]. Overexpression of NRP1 in renal or breast cancer cells enhances the Ras/ERK signaling [68], and that in pancreatic cancer cells induces chemoresistance [69]. PlGF also induces ERK1/2 phosphorylation through NRP1 and causes tumor growth and spread in medulloblastoma [70]. These lines of evidence indicate that NRPs play important roles in tumorigenesis and tumor progression through Rho-related signaling pathways.

**Figure 1:**
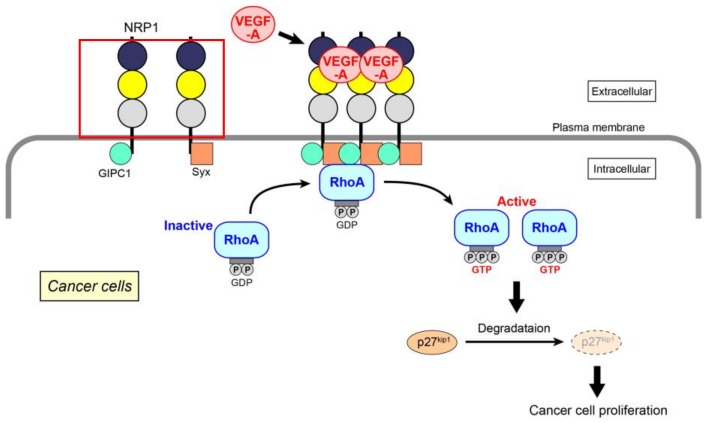
VEGF-A-initiated RhoA activation through NRP1 for cancer cell proliferation. After the binding of VEGF-A to NRP1, assembly of NRP1 occurs, resulting in increased GIPC1–Syx complex formation. RhoA is activated by Syx, a GEF for RhoA. Degradation of p27^kip1^ by activated RhoA contributes to the proliferation of cancer cells.

Extending the above data, we have recently identified the molecular mechanism of VEGF-A-induced RhoA activation for the proliferation of skin cancer, prostate cancer, and glioblastoma cells (Figure 1) [61]. Because the expression of VEGF receptors (both VEGF receptor-1 and -2) is almost absent in these cancer cells, the mechanism mainly depends on NRP1. When VEGF-A binds to NRP1, it facilitates the interaction of NRP1 with GIPC1, a scaffold protein. The assembly of a molecular complex of GIPC1 and Syx, a guanine nucleotide exchange factor (GEF) for RhoA, is then enhanced, leading to an increase in the GTP-bound active form of RhoA. This is the first demonstration that the activation of RhoA is induced by NRP1 alone in cancer cells, although RhoA in endothelial cells has been reported to be activated specifically by NRP1 through the Gq and PI3 kinase pathway for cell migration [71]. Activated RhoA contributes to the degradation of p27^kip1^ to promote cell proliferation. This RhoA activation signal is not affected by Rho-related signaling molecules, such as MAP kinases and Akt. There have been several studies that investigate the role of GIPC1 and Syx in cancer cells. GIPC1 has anti-apoptotic effects in human breast and colorectal cancer cells [72], and the interaction of GIPC1 with MyoGEF, a GEF for RhoA, activates RhoA and promotes breast cancer invasion [73]. Syx controls the spatiotemporal regulation of mDia1 and ROCK activities through RhoA in migrating glioblastoma multiforme (U251) and mammary grand tumor (Hs578T) cells [74]. Maintenance of epithelial cell polarity and the epithelial barrier at cell–cell junctions relies on the spatial organization of the actin cytoskeleton and proper positioning/assembly of intercellular junction complexes [39]. However, in tumor cells, epithelial–mesenchymal transition, which is a typical morphological change associated with the loss of cell polarity, often occurs together with RhoA activation [75]. The effectors of RhoA, ROCK, and mDia1 promote this effect through enhanced actin polymerization, resulting in the dissociation of cell junctions to induce tumor invasion.

In a tumor microenvironment, the exposure of endothelial cells to a high level of VEGF-A alters RhoGEF expression profiles [76]. Tumor-derived capillary endothelial cells harbor a high level of active RhoA and ROCK to exhibit aberrant vessel formation through mechanosensing abnormalities at the interface between endothelial cells and the extracellular matrix [77]. This suggests that the VEGF-A–RhoA pathway is a promising target for anticancer therapy. Diffuse-type gastric carcinomas reportedly possess mutations at Arg5, Gly17, and Tyr42 of RhoA, which cause RhoA gain-of-function and a poor prognosis [78,79]. Inhibition of NRP1 expression by miRNA-338 attenuates gastric cancer cell growth, migration, and invasion by blocking activation of the Rho-related molecules, ERK and p38 MAP kinase [80], suggesting the potential of the miRNA for antitumor therapeutics.

## 4. VEGF-A-Induced Functions of Rap in Angiogenesis

Similar to Rho, Rap is highly evolutionarily conserved with Ras [11]. Although the Rap subfamily includes Rap1 (Rap1a and Rap1b) and Rap2 (Rap2a, Rap2b and Rap2c) [81], we discuss only Rap1 in this article. The Rap1 signaling machinery relies on organism/species-specific effectors to regulate various cellular events [82]. Two isoforms of Rap1 protein have been identified: Rap1a and Rap1b. They share 95% amino acid identity, although they are encoded by two separate genes, *RAP1A*, and *RAP1B* [83]. Despite its name, the biological roles of Rap1 are considered to be independent of Ras signaling, owing to the availability of a distinct set of Rap1 regulatory and effector proteins [83]. Rap1 is involved in various cellular processes, including the organization of intercellular adhesion complexes, and cell proliferation, migration, and polarity [84,85,86]. Activated by GEFs, Rap1 forms “signalosomes” that mediate a broad range of physiological functions in the cardiovascular, nervous, endocrine, and respiratory systems [87].

Rap1 is of vital importance in angiogenesis. Genetic studies show that the deletion of either Rap1a or Rap1b in mice leads to partial embryonic lethality and defective angiogenic responses in surviving animals [88,89,90,91,92]. Rap1 functions downstream of the main angiogenic growth-controlling receptors in endothelial cells: fibroblast growth factor (FGF) receptor-1, VEGF receptor-2, and sphingosine 1-phosphate (S1P) receptors [92,93]. In addition, Rap1 exerts upstream effects on VEGF receptor-2 by promoting its activation through integrin α_v_β_3_, a molecule that is also required for VEGF-induced angiogenesis [94,95]. Rap1, in an isoform-specific manner, is also capable of regulating endothelial barrier permeability by VEGF stimulation [96]. The importance of Rap1 in angiogenic signaling related to VEGF, FGF, and S1P has been unequivocally confirmed, and Rap1 activation by various GEFs has been reported. However, the exact mechanisms of Rap1 regulation through the above angiogenic receptors have not yet been completely identified. Until recently, moreover, Rap1 effectors in angiogenic growth of the endothelium have been poorly understood.

**Figure 2:**
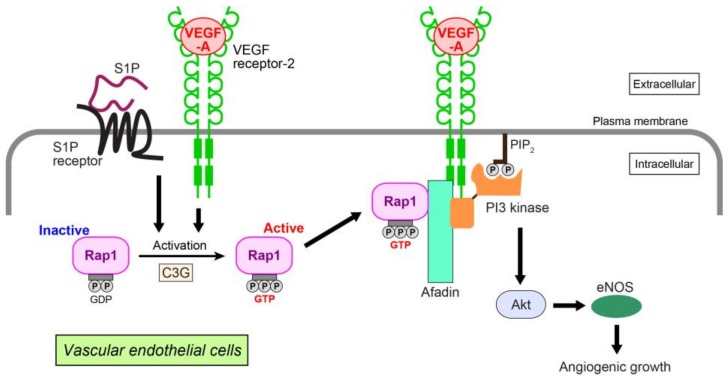
Angiogenic growth induced by VEGF-A or S1P in vascular endothelial cells. VEGF-A and S1P activate Rap1 through a Rap1-GEF C3G by binding to their cognate receptors. Activated Rap1 interacts with afadin, an adaptor protein, and recruits afadin and PI3 kinase to the plasma membrane of the leading edge, where VEGF receptor-2 associates with these molecules. Akt and eNOS are then activated, enhancing the angiogenic growth of endothelial cells. PIP_2_: phosphatidylinositol (4,5)-bisphosphate.

We have identified Rap1-related signaling events in VEGF-A- and S1P-induced angiogenic growth (Figure 2) [93]. In human umbilical vein endothelial cells (HUVECs), VEGF-A and/or S1P activate Rap1. This enhances interaction with afadin, an actin-binding adaptor protein having diverse cardiovascular functions [97,98,99]. The interaction alters the cellular localization of afadin, recruiting this molecule to the plasma membrane to interact with VEGF receptor-2. In polarized HUVECs, Rap1, C3G (a GEF for Rap1), afadin, and VEGF receptor-2 or S1P receptor colocalize at the leading edge of the cells, promoting cell migration, proliferation, and capillary-like network formation. These cellular events are mediated by organized localization of the cell adhesion molecules, VE-cadherin, claudin-5, nectin-2, and junctional adhesion molecule-A together with cortical F-actin. In HUVECs, Rap1 and afadin specifically control Akt/eNOS phosphorylation related to the VEGF-A or S1P angiogenic pathway [100,101], but not to ERK1/2 or p38 MAP kinase. More precisely, Rap1 and afadin facilitate interaction of the p85 regulatory subunit of PI3 kinase with VEGF receptor-2 for PI3 kinase activation. PI3 kinase is a key mediator of Akt-related VEGF-A- or S1P-induced angiogenic growth [102,103]. Taken together, these data suggest that mutual collaboration between GTP-bound Rap1 and afadin downstream of activated VEGF receptor-2 and S1P receptor regulates endothelial cell growth, migration, proliferation, and tubule formation by triggering Akt/eNOS signaling. An in vivo study using Rap1 knockout mice demonstrates that Rap1 increases newly formed vessels and blood perfusion during the revascularization phase of ischemic muscle injury induced by arterial ligation [88].

## 5. Mode of Association between VEGF-A and Rap1 at the Molecular Level

After the transmission of biochemical signals from VEGF-A–VEGF receptor-2 to Rap1, Rap1 enhances cell migration by interacting with Rap1-interacting adaptor molecule (RIAM), a member of the MRL protein family that also includes Mig-10 and lamellipodin [104], and with Rap1-binding molecule (RAPL), which resides on microtubules in the protruding edge of migrating endothelial cells [105]. These molecules may also participate in Rap1 activation in response to VEGF-A in a feedback manner. Integrins as well as VEGF receptor-2 are abundant at the protruding edge of migrating cells [106].

Integrins, such as integrin α_5_β_1_ and integrin α_v_β_3_, are tightly involved in endothelial cell migration, apoptotic protection, and angiogenic growth [106,107]. Integrins transmit the signaling in both intracellular and extracellular directions, respectively described as outside-in and inside-out signaling [108]. Outside-in-activated integrins aggregate and assemble cytosolic signaling complexes to regulate basic cellular functions, whereas inside-out activation modulates integrin affinity for extracellular matrix components [109]. Integrin α_v_β_3_ is up-regulated and forms complexes with VEGF receptor-2 in the process of angiogenic growth, promoting the kinase activity of the receptor [95]. In contrast, suppression of the adhesive properties of integrin α_v_β_3_ inhibits angiogenesis. Rap1 is one of the main molecules that mediate integrin activation, and Rap1 down-regulation prevents integrin conformational changes that are required for their activation in endothelial cells [88,110]. However, the precise mechanism of this effect has not yet been clarified. RAPL, RIAM, and other MRL adaptor proteins may link Rap1 with integrins [104,111,112]. Another possible intermediate molecule for integrin activation is Krev interaction trapped protein-1 (KRIT1)/cerebral cavernous malformation 1 (CCM1), of which autosomal dominant loss-of-function mutation causes CCM, because of compromised junctional integrity in endothelial cells [113]. KRIT1/CCM1 interacts with Rap1 via its band4.1/ezrin/radixin/moesin domain and stabilizes endothelial intercellular junctions by activation of integrin. Rap1 is also engaged in VEGF-A-induced endothelial cell migration by a mechanism that includes a non-receptor tyrosine kinase Bmx/Etk, which binds to activated Rap1 and is coupled to integrin signaling [114,115,116].

## 6. VEGF-A and Rap1 in Pathological Conditions

Retinal neovascularization derived from choroidal endothelial cells and macular edema consequent to increased permeability of the blood–retinal barrier is a leading cause of blindness in the course of age-related macular degeneration, diabetic retinopathy, and central retinal vein occlusion [117]. VEGF signaling has been proven to be a damaging factor in the above conditions, and thus, anti-VEGF agents are now one of the available therapeutic tools [118]. In retinal endothelial cells, Epac1-mediated Rap1 activation opposes VEGF-A- and tumor necrosis factor-α-induced consequences of excessive endothelial permeability through inhibition of ERK1/2 [119]. Rap1 also contributes to the organization and protection of endothelial cell tight junctions. How angiogenesis and neovascularization are regulated by VEGF-A-initiated activation of Rap1 is different in physiological versus pathological conditions. In addition, retinal pigment epithelium-associated VEGF-A stimulates transmigration of choroidal endothelial cells during the neovascularization process by activating Rac1 [120]. Activated Rap1 abolishes Rac1 activation and neovascularization by reducing ROS generation [121]. These reports point to Epac/Rap1 as an alternative pharmacological target in retinal neovascularization syndromes.

Signaling directions inside cells may differ between physiological and pathological pathways [122]. In tumor cells, Rap1 positively or negatively regulates VEGF-A expression dependent on the environment surrounding the tumor [123,124]. One example is VEGF-A expression in pulmonary neuroendocrine tumor cells. In these cells, the interaction of a cytoskeletal protein filamin-A with Rap1 is up-regulated to promote cell migration and to decrease cell adhesion, resulting in increased malignant potential of the tumor cells [125,126]. Thus, VEGF-A–VEGF receptor signaling supports tumor expansion not only through new vessel growth via Rap, but also by directly regulating the cancer cells [94,127].

## 7. Conclusions

In summary, the small G proteins Rho and Rap and their regulatory molecules and effectors participate in physiologically morphogenetic and cellular events such as cell movement, proliferation, polarization, and adhesion. These events are necessary not only for angiogenesis in the cardiovascular system, but also for development and maintenance of various organs. In response to VEGF-A, Rho and Rap function as “molecular switches” that can mediate complicated crosstalk between various molecules and produce differential effects. Rho and Rap are also involved in VEGF-A-triggered pathological conditions, such as abnormal angiogenesis and tumor progression. Thus, targeting VEGF-A and its related molecules, including Rho and Rap, has significant therapeutic potential. However, the clinical outcome of anti-VEGF/VEGF receptor therapy is still far from satisfactory [128]. Because Rho and Rap are key components of the VEGF-A-induced signaling machinery, they may become new pharmacological targets for treating pathological disorders and diseases. In clinical practice and in researches, there are working examples of medications that interfere with the activation of the small G proteins and their related pathways [129,130,131]. However, it should be considered that some adverse/collateral effects might occur by targeting Rho and Rap for pharmacological intervention, because the small G proteins are cross points downstream of different membrane receptors.

## Abbreviations

VEGF	vascular endothelial growth factor
PlGF	placental growth factor
Rho	Ras homologue gene
Rap	Ras-related protein
ERK	extracellular signal-regulated kinase
PI3	phosphoinositide-3
PIP_3_	phosphatidylinositol (3,4,5)-trisphosphate
eNOS	endothelial nitric oxide synthase
MAP	mitogen-activated protein
ROS	reactive oxygen species
ROCK	Rho kinase
MLC	myosin light chain
NRP	neuropilin
GEF	guanine nucleotide exchange factor
FGF	fibroblast growth factor
S1P	sphingosine 1-phosphate
HUVEC	human umbilical vein endothelial cell
CCM	cerebral cavernous malformation

## References

[1] Chung A.S., Ferrara N. (2011). Developmental and pathological angiogenesis. Annu. Rev. Cell Dev. Biol..

[2] Simons M., Gordon E., Claesson-Welsh L. (2016). Mechanisms and regulation of endothelial VEGF receptor signalling. Nat. Rev. Mol. Cell Biol..

[3] Ferrara N., Carver-Moore K., Chen H., Dowd M., Lu L., O’Shea K.S., Powell-Braxton L., Hillan K.J., Moore M.W. (1996). Heterozygous embryonic lethality induced by targeted inactivation of the VEGF gene. Nature.

[4] Carmeliet P., Ferreira V., Breier G., Pollefeyt S., Kieckens L., Gertsenstein M., Fahrig M., Vandenhoeck A., Harpal K., Eberhardt C. (1996). Abnormal blood vessel development and lethality in embryos lacking a single VEGF allele. Nature.

[5] Hanahan D., Folkman J. (1996). Patterns and emerging mechanisms of the angiogenic switch during tumorigenesis. Cell.

[6] Kerbel R., Folkman J. (2002). Clinical translation of angiogenesis inhibitors. Nat. Rev. Cancer.

[7] Shibuya M. (2013). Vascular endothelial growth factor and its receptor system: Physiological functions in angiogenesis and pathological roles in various diseases. J. Biochem..

[8] Van der Meel R., Symons M.H., Kudernatsch R., Kok R.J., Schiffelers R.M., Storm G., Gallagher W.M., Byrne A.T. (2011). The VEGF/Rho GTPase signalling pathway: A promising target for anti-angiogenic/anti-invasion therapy. Drug Discov. Today.

[9] Heasman S.J., Ridley A.J. (2008). Mammalian Rho GTPases: New insights into their functions from in vivo studies. Nat. Rev. Mol. Cell Biol..

[10] Loirand G., Sauzeau V., Pacaud P. (2013). Small G proteins in the cardiovascular system: Physiological and pathological aspects. Physiol. Rev..

[11] Rojas A.M., Fuentes G., Rausell A., Valencia A. (2012). The Ras protein superfamily: Evolutionary tree and role of conserved amino acids. J. Cell Biol..

[12] Cross M.J., Dixelius J., Matsumoto T., Claesson-Welsh L. (2003). VEGF-receptor signal transduction. Trends Biochem. Sci..

[13] Takahashi T., Ueno H., Shibuya M. (1999). VEGF activates protein kinase C-dependent, but Ras-independent Raf-MEK-MAP kinase pathway for DNA synthesis in primary endothelial cells. Oncogene.

[14] Gerber H.P., McMurtrey A., Kowalski J., Yan M., Keyt B.A., Dixit V., Ferrara N. (1998). Vascular endothelial growth factor regulates endothelial cell survival through the phosphatidylinositol 3′-kinase/Akt signal transduction pathway. Requirement for Flk-1/KDR activation. J. Biol. Chem..

[15] Daher Z., Boulay P.L., Desjardins F., Gratton J.P., Claing A. (2010). Vascular endothelial growth factor receptor-2 activates ADP-ribosylation factor 1 to promote endothelial nitric-oxide synthase activation and nitric oxide release from endothelial cells. J. Biol. Chem..

[16] Takahashi T., Yamaguchi S., Chida K., Shibuya M. (2001). A single autophosphorylation site on KDR/Flk-1 is essential for VEGF-A-dependent activation of PLC-γ and DNA synthesis in vascular endothelial cells. EMBO J..

[17] Eliceiri B.P., Paul R., Schwartzberg P.L., Hood J.D., Leng J., Cheresh D.A. (1999). Selective requirement for Src kinases during VEGF-induced angiogenesis and vascular permeability. Mol. Cell.

[18] Qi J.H., Claesson-Welsh L. (2001). VEGF-induced activation of phosphoinositide 3-kinase is dependent on focal adhesion kinase. Exp. Cell Res..

[19] Rousseau S., Houle F., Landry J., Huot J. (1997). p38 MAP kinase activation by vascular endothelial growth factor mediates actin reorganization and cell migration in human endothelial cells. Oncogene.

[20] Lu Q., Rounds S. (2012). Focal adhesion kinase and endothelial cell apoptosis. Microvasc. Res..

[21] Nuche-Berenguer B., Ramos-Álvarez I., Jensen R.T. (2016). The p21-activated kinase, PAK2, is important in the activation of numerous pancreatic acinar cell signaling cascades and in the onset of early pancreatitis events. Biochim. Biophys. Acta.

[22] Sabbatini M., Santillo M., Pisani A., Paternò R., Uccello F., Serù R., Matrone G., Spagnuolo G., Andreucci M., Serio V. (2006). Inhibition of Ras/ERK1/2 signaling protects against postischemic renal injury. Am. J. Physiol. Renal Physiol..

[23] Witte D., Bartscht T., Kaufmann R., Pries R., Settmacher U., Lehnert H., Ungefroren H. (2017). TGF-β1-induced cell migration in pancreatic carcinoma cells is RAC1 and NOX4-dependent and requires RAC1 and NOX4-dependent activation of p38 MAPK. Oncol. Rep..

[24] Bates D.O. (2010). Vascular endothelial growth factors and vascular permeability. Cardiovasc. Res..

[25] Beckers C.M., van Hinsbergh V.W., van Nieuw Amerongen G.P. (2010). Driving Rho GTPase activity in endothelial cells regulates barrier integrity. Thromb. Haemost..

[26] Eriksson A., Cao R., Roy J., Tritsaris K., Wahlestedt C., Dissing S., Thyberg J., Cao Y. (2003). Small GTP-binding protein Rac is an essential mediator of vascular endothelial growth factor-induced endothelial fenestrations and vascular permeability. Circulation.

[27] Monaghan-Benson E., Burridge K. (2009). The regulation of vascular endothelial growth factor-induced microvascular permeability requires Rac and reactive oxygen species. J. Biol. Chem..

[28] Ogita H., Liao J.K. (2004). Endothelial function and oxidative stress. Endothelium.

[29] Yamaoka-Tojo M., Ushio-Fukai M., Hilenski L., Dikalov S.I., Chen Y.E., Tojo T., Fukai T., Fujimoto M., Patrushev N.A., Wang N. (2004). IQGAP1, a novel vascular endothelial growth factor receptor binding protein, is involved in reactive oxygen species—Dependent endothelial migration and proliferation. Circ. Res..

[30] Tan W., Palmby T.R., Gavard J., Amornphimoltham P., Zheng Y., Gutkind J.S. (2008). An essential role for Rac1 in endothelial cell function and vascular development. FASEB J..

[31] Lamalice L., Houle F., Jourdan G., Huot J. (2004). Phosphorylation of tyrosine 1214 on VEGFR2 is required for VEGF-induced activation of Cdc42 upstream of SAPK2/p38. Oncogene.

[32] Engelse M.A., Laurens N., Verloop R.E., Koolwijk P., van Hinsbergh V.W. (2008). Differential gene expression analysis of tubule forming and non-tubule forming endothelial cells: CDC42GAP as a counter-regulator in tubule formation. Angiogenesis.

[33] Hu G., Chen Y., Zhang L., Tong W., Cheng Y., Luo Y., Cai S., Zhang L. (2011). The generation of the endothelial specific Cdc42-deficient mice and the effect of Cdc42 deletion on the angiogenesis and embryonic development. Chin. Med. J..

[34] Barry D.M., Xu K., Meadows S.M., Zheng Y., Norden P.R., Davis G.E., Cleaver O. (2015). Cdc42 is required for cytoskeletal support of endothelial cell adhesion during blood vessel formation in mice. Development.

[35] Sun H., Breslin J.W., Zhu J., Yuan S.Y., Wu M.H. (2006). Rho and ROCK signaling in VEGF-induced microvascular endothelial hyperpermeability. Microcirculation.

[36] Cerutti C., Ridley A.J. (2017). Endothelial cell-cell adhesion and signaling. Exp. Cell Res..

[37] Dejana E. (2004). Endothelial cell-cell junctions: Happy together. Nat. Rev. Mol. Cell Biol..

[38] Dejana E., Orsenigo F., Molendini C., Baluk P., McDonald D.M. (2009). Organization and signaling of endothelial cell-to-cell junctions in various regions of the blood and lymphatic vascular trees. Cell Tissue Res..

[39] Campos Y., Qiu X., Gomero E., Wakefield R., Horner L., Brutkowski W., Han Y.G., Solecki D., Frase S., Bongiovanni A. (2016). Alix-mediated assembly of the actomyosin-tight junction polarity complex preserves epithelial polarity and epithelial barrier. Nat. Commun..

[40] Van Nieuw Amerongen G.P., Koolwijk P., Versteilen A., van Hinsbergh V.W. (2003). Involvement of RhoA/Rho kinase signaling in VEGF-induced endothelial cell migration and angiogenesis in vitro. Arterioscler. Thromb. Vasc. Biol..

[41] Mammoto A., Huang S., Moore K., Oh P., Ingber D.E. (2004). Role of RhoA, mDia, and ROCK in cell shape-dependent control of the Skp2-p27kip1 pathway and the G1/S transition. J. Biol. Chem..

[42] Glotzer M. (2001). Animal cell cytokinesis. Annu. Rev. Cell Dev. Biol..

[43] Yamaguchi M., Nakao S., Arima M., Wada I., Kaizu Y., Hao F., Yoshida S., Sonoda K.H. (2017). Rho-Kinase/ROCK as a Potential Drug Target for Vitreoretinal Diseases. J. Ophthalmol..

[44] Bolinger M.T., Antonetti D.A. (2016). Moving Past Anti-VEGF: Novel Therapies for Treating Diabetic Retinopathy. Int. J. Mol. Sci..

[45] Inoue M., Hager J.H., Ferrara N., Gerber H.P., Hanahan D. (2002). VEGF-A has a critical, nonredundant role in angiogenic switching and pancreatic β cell carcinogenesis. Cancer Cell.

[46] Mirones I., Conti C.J., Martínez J., Garcia M., Larcher F. (2009). Complexity of VEGF responses in skin carcinogenesis revealed through ex vivo assays based on a VEGF-A null mouse keratinocyte cell line. J. Investig. Dermatol..

[47] Park S.T., Kim B.R., Park S.H., Lee J.H., Lee E.J., Lee S.H., Rho S.B. (2014). Suppression of VEGF expression through interruption of the HIF-1α and Akt signaling cascade modulates the anti-angiogenic activity of DAPK in ovarian carcinoma cells. Oncol. Rep..

[48] Chen S., Wang J., Gou W.F., Xiu Y.L., Zheng H.C., Zong Z.H., Takano Y., Zhao Y. (2013). The involvement of RhoA and Wnt-5a in the tumorigenesis and progression of ovarian epithelial carcinoma. Int. J. Mol. Sci..

[49] Xue Y., Bi F., Zhang X., Zhang S., Pan Y., Liu N., Shi Y., Yao X., Zheng Y., Fan D. (2006). Role of Rac1 and Cdc42 in hypoxia induced p53 and von Hippel-Lindau suppression and HIF1α activation. Int. J. Cancer.

[50] Fan F., Wey J.S., McCarty M.F., Belcheva A., Liu W., Bauer T.W., Somcio R.J., Wu Y., Hooper A., Hicklin D.J. (2005). Expression and function of vascular endothelial growth factor receptor-1 on human colorectal cancer cells. Oncogene.

[51] Bates R.C., Goldsmith J.D., Bachelder R.E., Brown C., Shibuya M., Oettgen P., Mercurio A.M. (2003). Flt-1-dependent survival characterizes the epithelial-mesenchymal transition of colonic organoids. Curr. Biol..

[52] Su J.L., Yang P.C., Shih J.Y., Yang C.Y., Wei L.H., Hsieh C.Y., Chou C.H., Jeng Y.M., Wang M.Y., Chang K.J. (2006). The VEGF-C/Flt-4 axis promotes invasion and metastasis of cancer cells. Cancer Cell.

[53] Aesoy R., Sanchez B.C., Norum J.H., Lewensohn R., Viktorsson K., Linderholm B. (2008). An autocrine VEGF/VEGFR2 and p38 signaling loop confers resistance to 4-hydroxytamoxifen in MCF-7 breast cancer cells. Mol. Cancer Res..

[54] Oommen S., Gupta S.K., Vlahakis N.E. (2011). Vascular endothelial growth factor A (VEGF-A) induces endothelial and cancer cell migration through direct binding to integrin α9β1: Identification of a specific α9β1 binding site. J. Biol. Chem..

[55] Luo M., Hou L., Li J., Shao S., Huang S., Meng D., Liu L., Feng L., Xia P., Qin T. (2016). VEGF/NRP-1axis promotes progression of breast cancer via enhancement of epithelial-mesenchymal transition and activation of NF-κB and β-catenin. Cancer Lett..

[56] Wang J., Huang Y., Zhang J., Xing B., Xuan W., Wang H., Huang H., Yang J., Tang J. (2018). NRP-2 in tumor lymphangiogenesis and lymphatic metastasis. Cancer Lett..

[57] Geretti E., Shimizu A., Klagsbrun M. (2008). Neuropilin structure governs VEGF and semaphorin binding and regulates angiogenesis. Angiogenesis.

[58] Smith N.R., Baker D., James N.H., Ratcliffe K., Jenkins M., Ashton S.E., Sproat G., Swann R., Gray N., Ryan A. (2010). Vascular endothelial growth factor receptors VEGFR-2 and VEGFR-3 are localized primarily to the vasculature in human primary solid cancers. Clin. Cancer Res..

[59] Zhang L., Wang H., Li C., Zhao Y., Wu L., Du X., Han Z. (2017). VEGF-A/Neuropilin 1 Pathway Confers Cancer Stemness via Activating Wnt/β-Catenin Axis in Breast Cancer Cells. Cell. Physiol. Biochem..

[60] Atzori M.G., Tentori L., Ruffini F., Ceci C., Lisi L., Bonanno E., Scimeca M., Eskilsson E., Daubon T., Miletic H. (2017). The anti-vascular endothelial growth factor receptor-1 monoclonal antibody D16F7 inhibits invasiveness of human glioblastoma and glioblastoma stem cells. J. Exp. Clin. Cancer Res..

[61] Yoshida A., Shimizu A., Asano H., Kadonosono T., Kondoh S.K., Geretti E., Mammoto A., Klagsbrun M., Seo M.K. (2015). VEGF-A/NRP1 stimulates GIPC1 and Syx complex formation to promote RhoA activation and proliferation in skin cancer cells. Biol. Open.

[62] Chen L., Miao W., Tang X., Zhang H., Wang S., Luo F., Yan J. (2013). Inhibitory effect of neuropilin-1 monoclonal antibody (NRP-1 MAb) on glioma tumor in mice. J. Biomed. Nanotechnol..

[63] Glinka Y., Mohammed N., Subramaniam V., Jothy S., Prud’homme G.J. (2012). Neuropilin-1 is expressed by breast cancer stem-like cells and is linked to NF-κB activation and tumor sphere formation. Biochem. Biophys. Res. Commun..

[64] Pan Q., Chanthery Y., Liang W.C., Stawicki S., Mak J., Rathore N., Tong R.K., Kowalski J., Yee S.F., Pacheco G. (2007). Blocking neuropilin-1 function has an additive effect with anti-VEGF to inhibit tumor growth. Cancer Cell.

[65] Nguyen Q.D., Rodrigues S., Rodrigue C.M., Rivat C., Grijelmo C., Bruyneel E., Emami S., Attoub S., Gespach C. (2006). Inhibition of vascular endothelial growth factor (VEGF)-165 and semaphorin 3A-mediated cellular invasion and tumor growth by the VEGF signaling inhibitor ZD4190 in human colon cancer cells and xenografts. Mol. Cancer Ther..

[66] Shimizu A., Mammoto A., Italiano J.E., Pravda E., Dudley A.C., Ingber D.E., Klagsbrun M. (2008). ABL2/ARG tyrosine kinase mediates SEMA3F-induced RhoA inactivation and cytoskeleton collapse in human glioma cells. J. Biol. Chem..

[67] Bachelder R.E., Crago A., Chung J., Wendt M.A., Shaw L.M., Robinson G., Mercurio A.M. (2001). Vascular endothelial growth factor is an autocrine survival factor for neuropilin-expressing breast carcinoma cells. Cancer Res..

[68] Cao Y., E G., Wang E., Pal K., Dutta S.K., Bar-Sagi D., Mukhopadhyay D. (2012). VEGF exerts an angiogenesis-independent function in cancer cells to promote their malignant progression. Cancer Res..

[69] Wey J.S., Gray M.J., Fan F., Belcheva A., McCarty M.F., Stoeltzing O., Somcio R., Liu W., Evans D.B., Klagsbrun M. (2005). Overexpression of neuropilin-1 promotes constitutive MAPK signalling and chemoresistance in pancreatic cancer cells. Br. J. Cancer.

[70] Snuderl M., Batista A., Kirkpatrick N.D., Ruiz de Almodovar C., Riedemann L., Walsh E.C., Anolik R., Huang Y., Martin J.D., Kamoun W. (2013). Targeting placental growth factor/neuropilin 1 pathway inhibits growth and spread of medulloblastoma. Cell.

[71] Wang L., Zeng H., Wang P., Soker S., Mukhopadhyay D. (2003). Neuropilin-1-mediated vascular permeability factor/vascular endothelial growth factor-dependent endothelial cell migration. J. Biol. Chem..

[72] Chittenden T.W., Pak J., Rubio R., Cheng H., Holton K., Prendergast N., Glinskii V., Cai Y., Culhane A., Bentink S. (2010). Therapeutic implications of GIPC1 silencing in cancer. PLoS ONE.

[73] Wu D., Haruta A., Wei Q. (2010). GIPC1 interacts with MyoGEF and promotes MDA-MB-231 breast cancer cell invasion. J. Biol. Chem..

[74] Dachsel J.C., Ngok S.P., Lewis-Tuffin L.J., Kourtidis A., Geyer R., Johnston L., Feathers R., Anastasiadis P.Z. (2013). The Rho guanine nucleotide exchange factor Syx regulates the balance of Dia and ROCK activities to promote polarized-cancer-cell migration. Mol. Cell. Biol..

[75] Lamouille S., Xu J., Derynck R. (2014). Molecular mechanisms of epithelial-mesenchymal transition. Nat. Rev. Mol. Cell Biol..

[76] Hernández-García R., Iruela-Arispe M.L., Reyes-Cruz G., Vázquez-Prado J. (2015). Endothelial RhoGEFs: A systematic analysis of their expression profiles in VEGF-stimulated and tumor endothelial cells. Vasc. Pharmacol..

[77] Ghosh K., Thodeti C.K., Dudley A.C., Mammoto A., Klagsbrun M., Ingber D.E. (2008). Tumor-derived endothelial cells exhibit aberrant Rho-mediated mechanosensing and abnormal angiogenesis in vitro. Proc. Natl. Acad. Sci. USA.

[78] Kakiuchi M., Nishizawa T., Ueda H., Gotoh K., Tanaka A., Hayashi A., Yamamoto S., Tatsuno K., Katoh H., Watanabe Y. (2014). Recurrent gain-of-function mutations of RHOA in diffuse-type gastric carcinoma. Nat. Genet..

[79] Kwon C.H., Kim Y.K., Lee S., Kim A., Park H.J., Choi Y., Won Y.J., Park D.Y., Lauwers G.Y. (2018). Gastric poorly cohesive carcinoma: A correlative study of mutational signatures and prognostic significance based on histopathological subtypes. Histopathology.

[80] Peng Y., Liu Y.M., Li L.C., Wang L.L., Wu X.L. (2014). MicroRNA-338 inhibits growth, invasion and metastasis of gastric cancer by targeting NRP1 expression. PLoS ONE.

[81] Minato N. (2013). Rap G protein signal in normal and disordered lymphohematopoiesis. Exp. Cell Res..

[82] Frische E.W., Zwartkruis F.J. (2010). Rap1, a mercenary among the Ras-like GTPases. Dev. Biol..

[83] Pizon V., Chardin P., Lerosey I., Olofsson B., Tavitian A. (1988). Human cDNAs rap1 and rap2 homologous to the Drosophila gene Dras3 encode proteins closely related to ras in the ‘effector’ region. Oncogene.

[84] Altschuler D.L., Ribeiro-Neto F. (1998). Mitogenic and oncogenic properties of the small G protein Rap1b. Proc. Natl. Acad. Sci. USA.

[85] Pannekoek W.J., Kooistra M.R., Zwartkruis F.J., Bos J.L. (2009). Cell-cell junction formation: The role of Rap1 and Rap1 guanine nucleotide exchange factors. Biochim. Biophys. Acta.

[86] Wilson C.W., Ye W. (2014). Regulation of vascular endothelial junction stability and remodeling through Rap1-Rasip1 signaling. Cell Adhes. Migr..

[87] Schmidt M., Dekker F.J., Maarsingh H. (2013). Exchange protein directly activated by cAMP (epac): A multidomain cAMP mediator in the regulation of diverse biological functions. Pharmacol. Rev..

[88] Carmona G., Göttig S., Orlandi A., Scheele J., Bäuerle T., Jugold M., Kiessling F., Henschler R., Zeiher A.M., Dimmeler S. (2009). Role of the small GTPase Rap1 for integrin activity regulation in endothelial cells and angiogenesis. Blood.

[89] Chrzanowska-Wodnicka M., Smyth S.S., Schoenwaelder S.M., Fischer T.H., White G.C. (2005). Rap1b is required for normal platelet function and hemostasis in mice. J. Clin. Investig..

[90] Chrzanowska-Wodnicka M., Kraus A.E., Gale D., White G.C., Vansluys J. (2008). Defective angiogenesis, endothelial migration, proliferation, and MAPK signaling in Rap1b-deficient mice. Blood.

[91] Li Y., Yan J., De P., Chang H.C., Yamauchi A., Christopherson K.W., Paranavitana N.C., Peng X., Kim C., Munugalavadla V. (2007). Rap1a null mice have altered myeloid cell functions suggesting distinct roles for the closely related Rap1a and 1b proteins. J. Immunol..

[92] Yan J., Li F., Ingram D.A., Quilliam L.A. (2008). Rap1a is a key regulator of fibroblast growth factor 2-induced angiogenesis and together with Rap1b controls human endothelial cell functions. Mol. Cell. Biol..

[93] Tawa H., Rikitake Y., Takahashi M., Amano H., Miyata M., Satomi-Kobayashi S., Kinugasa M., Nagamatsu Y., Majima T., Ogita H. (2010). Role of afadin in vascular endothelial growth factor- and sphingosine 1-phosphate-induced angiogenesis. Circ. Res..

[94] Lakshmikanthan S., Sobczak M., Chun C., Henschel A., Dargatz J., Ramchandran R., Chrzanowska-Wodnicka M. (2011). Rap1 promotes VEGFR2 activation and angiogenesis by a mechanism involving integrin αvβ3. Blood.

[95] Somanath P.R., Malinin N.L., Byzova T.V. (2009). Cooperation between integrin αvβ3 and VEGFR2 in angiogenesis. Angiogenesis.

[96] Lakshmikanthan S., Sobczak M., Li Calzi S., Shaw L., Grant M.B., Chrzanowska-Wodnicka M. (2018). Rap1B promotes VEGF-induced endothelial permeability and is required for dynamic regulation of the endothelial barrier. J. Cell Sci..

[97] Majima T., Takeuchi K., Sano K., Hirashima M., Zankov D.P., Tanaka-Okamoto M., Ishizaki H., Miyoshi J., Ogita H. (2013). An Adaptor Molecule Afadin Regulates Lymphangiogenesis by Modulating RhoA Activity in the Developing Mouse Embryo. PLoS ONE.

[98] Zankov D.P., Sato A., Shimizu A., Ogita H. (2017). Differential Effects of Myocardial Afadin on Pressure Overload-Induced Compensated Cardiac Hypertrophy. Circ. J..

[99] Zankov D.P., Shimizu A., Tanaka-Okamoto M., Miyoshi J., Ogita H. (2017). Protective effects of intercalated disk protein afadin on chronic pressure overload-induced myocardial damage. Sci. Rep..

[100] Ackah E., Yu J., Zoellner S., Iwakiri Y., Skurk C., Shibata R., Ouchi N., Easton R.M., Galasso G., Birnbaum M.J. (2005). Akt1/protein kinase Bα is critical for ischemic and VEGF-mediated angiogenesis. J. Clin. Investig..

[101] Rikitake Y., Hirata K., Kawashima S., Ozaki M., Takahashi T., Ogawa W., Inoue N., Yokoyama M. (2002). Involvement of endothelial nitric oxide in sphingosine-1-phosphate-induced angiogenesis. Arterioscler. Thromb. Vasc. Biol..

[102] Graupera M., Guillermet-Guibert J., Foukas L.C., Phng L.K., Cain R.J., Salpekar A., Pearce W., Meek S., Millan J., Cutillas P.R. (2008). Angiogenesis selectively requires the p110α isoform of PI3K to control endothelial cell migration. Nature.

[103] Heller R., Chang Q., Ehrlich G., Hsieh S.N., Schoenwaelder S.M., Kuhlencordt P.J., Preissner K.T., Hirsch E., Wetzker R. (2008). Overlapping and distinct roles for PI3Kβ and γ isoforms in S1P-induced migration of human and mouse endothelial cells. Cardiovasc. Res..

[104] Lafuente E.M., van Puijenbroek A.A., Krause M., Carman C.V., Freeman G.J., Berezovskaya A., Constantine E., Springer T.A., Gertler F.B., Boussiotis V.A. (2004). RIAM, an Ena/VASP and Profilin ligand, interacts with Rap1-GTP and mediates Rap1-induced adhesion. Dev. Cell.

[105] Fujita H., Fukuhara S., Sakurai A., Yamagishi A., Kamioka Y., Nakaoka Y., Masuda M., Mochizuki N. (2005). Local activation of Rap1 contributes to directional vascular endothelial cell migration accompanied by extension of microtubules on which RAPL, a Rap1-associating molecule, localizes. J. Biol. Chem..

[106] Avraamides C.J., Garmy-Susini B., Varner J.A. (2008). Integrins in angiogenesis and lymphangiogenesis. Nat. Rev. Cancer.

[107] Eliceiri B.P., Cheresh D.A. (1999). The role of αv integrins during angiogenesis: Insights into potential mechanisms of action and clinical development. J. Clin. Investig..

[108] Legate K.R., Wickström S.A., Fässler R. (2009). Genetic and cell biological analysis of integrin outside-in signaling. Genes Dev..

[109] Hynes R.O. (2002). Integrins: Bidirectional, allosteric signaling machines. Cell.

[110] Caron E. (2003). Cellular functions of the Rap1 GTP-binding protein: A pattern emerges. J. Cell Sci..

[111] Katagiri K., Maeda A., Shimonaka M., Kinashi T. (2003). RAPL, a Rap1-binding molecule that mediates Rap1-induced adhesion through spatial regulation of LFA-1. Nat. Immunol..

[112] Lagarrigue F., Kim C., Ginsberg M.H. (2016). The Rap1-RIAM-talin axis of integrin activation and blood cell function. Blood.

[113] Glading A., Han J., Stockton R.A., Ginsberg M.H. (2007). KRIT-1/CCM1 is a Rap1 effector that regulates endothelial cell cell junctions. J. Cell Biol..

[114] Abassi Y.A., Rehn M., Ekman N., Alitalo K., Vuori K. (2003). p130Cas Couples the tyrosine kinase Bmx/Etk with regulation of the actin cytoskeleton and cell migration. J. Biol. Chem..

[115] Stoletov K.V., Terman B.I. (2004). Bmx is a downstream Rap1 effector in VEGF-induced endothelial cell activation. Biochem. Biophys. Res. Commun..

[116] Tamagnone L., Lahtinen I., Mustonen T., Virtaneva K., Francis F., Muscatelli F., Alitalo R., Smith C.I., Larsson C., Alitalo K. (1994). BMX, a novel nonreceptor tyrosine kinase gene of the BTK/ITK/TEC/TXK family located in chromosome Xp22.2. Oncogene.

[117] Holz F.G., Schmitz-Valckenberg S., Fleckenstein M. (2014). Recent developments in the treatment of age-related macular degeneration. J. Clin. Investig..

[118] Cheung N., Wong I.Y., Wong T.Y. (2014). Ocular anti-VEGF therapy for diabetic retinopathy: Overview of clinical efficacy and evolving applications. Diabetes Care.

[119] Ramos C.J., Lin C., Liu X., Antonetti D.A. (2018). The EPAC-Rap1 pathway prevents and reverses cytokine-induced retinal vascular permeability. J. Biol. Chem..

[120] Wang H., Geisen P., Wittchen E.S., King B., Burridge K., D’Amore P.A., Hartnett M.E. (2011). The role of RPE cell-associated VEGF_189_ in choroidal endothelial cell transmigration across the RPE. Investig. Ophthalmol. Vis. Sci..

[121] Wang H., Fotheringham L., Wittchen E.S., Hartnett M.E. (2015). Rap1 GTPase Inhibits Tumor Necrosis Factor-α-Induced Choroidal Endothelial Migration via NADPH Oxidase- and NF-κB-Dependent Activation of Rac1. Am. J. Pathol..

[122] Dewerchin M., Carmeliet P. (2012). PlGF: A multitasking cytokine with disease-restricted activity. Cold Spring Harb. Perspect. Med..

[123] Menon J., Doebele R.C., Gomes S., Bevilacqua E., Reindl K.M., Rosner M.R. (2012). A novel interplay between Rap1 and PKA regulates induction of angiogenesis in prostate cancer. PLoS ONE.

[124] Sheta E.A., Harding M.A., Conaway M.R., Theodorescu D. (2000). Focal adhesion kinase, Rap1, and transcriptional induction of vascular endothelial growth factor. J. Natl. Cancer Inst..

[125] Uramoto H., Akyürek L.M., Hanagiri T. (2010). A positive relationship between filamin and VEGF in patients with lung cancer. Anticancer Res..

[126] Vitali E., Boemi I., Rosso L., Cambiaghi V., Novellis P., Mantovani G., Spada A., Alloisio M., Veronesi G., Ferrero S. (2017). FLNA is implicated in pulmonary neuroendocrine tumors aggressiveness and progression. Oncotarget.

[127] Dias S., Hattori K., Zhu Z., Heissig B., Choy M., Lane W., Wu Y., Chadburn A., Hyjek E., Gill M. (2000). Autocrine stimulation of VEGFR-2 activates human leukemic cell growth and migration. J. Clin. Investig..

[128] Sitohy B., Nagy J.A., Dvorak H.F. (2012). Anti-VEGF/VEGFR therapy for cancer: Reassessing the target. Cancer Res..

[129] Dong M., Yan B.P., Liao J.K., Lam Y.Y., Yip G.W., Yu C.M. (2010). Rho-kinase inhibition: A novel therapeutic target for the treatment of cardiovascular diseases. Drug Discov. Today.

[130] Mor A., Haklai R., Ben-Moshe O., Mekori Y.A., Kloog Y. (2011). Inhibition of contact sensitivity by farnesylthiosalicylic acid-amide, a potential Rap1 inhibitor. J. Investig. Dermatol..

[131] Ooshiro D., Yamaguchi S., Kakazu M., Arasaki O. (2017). Effectiveness of continuous low-dose fasudil on refractory coronary vasospasm subsequent to cardiopulmonary arrest. Clin. Case Rep..

